# Effects of Combined Ketamine/Xylazine Anesthesia on Light Induced Retinal Degeneration in Rats

**DOI:** 10.1371/journal.pone.0035687

**Published:** 2012-04-25

**Authors:** Blanca Arango-Gonzalez, Andreas Schatz, Sylvia Bolz, Javier Eslava-Schmalbach, Gabriel Willmann, Ahmad Zhour, Eberhart Zrenner, M. Dominik Fischer, Florian Gekeler

**Affiliations:** 1 Division of Experimental Ophthalmology, Centre for Ophthalmology, Tuebingen, Germany; 2 Clinical Research Institute, National University of Colombia, Bogota, Colombia; University of Florida, United States of America

## Abstract

**Objectives:**

To explore the effect of ketamine-xylazine anesthesia on light-induced retinal degeneration in rats.

**Methods:**

Rats were anesthetized with ketamine and xylazine (100 and 5 mg, respectively) for 1 h, followed by a recovery phase of 2 h before exposure to 16,000 lux of environmental illumination for 2 h. Functional assessment by electroretinography (ERG) and morphological assessment by *in vivo* imaging (optical coherence tomography), histology (hematoxylin/eosin staining, TUNEL assay) and immunohistochemistry (GFAP and rhodopsin staining) were performed at baseline (ERG), 36 h, 7 d and 14 d post-treatment. Non-anesthetized animals treated with light damage served as controls.

**Results:**

Ketamine-xylazine pre-treatment preserved retinal function and protected against light-induced retinal degeneration. *In vivo* retinal imaging demonstrated a significant increase of outer nuclear layer (ONL) thickness in the non-anesthetized group at 36 h (*p*<0.01) and significant reduction one week (*p*<0.01) after light damage. In contrast, ketamine-xylazine pre-treated animals showed no significant alteration of total retinal or ONL thickness at either time point (*p*>0.05), indicating a stabilizing and/or protective effect with regard to phototoxicity. Histology confirmed light-induced photoreceptor cell death and Müller cells gliosis in non-anesthetized rats, especially in the superior hemiretina, while ketamine-xylazine treated rats showed reduced photoreceptor cell death (TUNEL staining: *p*<0.001 after 7 d), thicker ONL and longer IS/OS. Fourteen days after light damage, a reduction of standard flash induced a-wave amplitudes and a-wave slopes (*p* = 0.01) and significant alterations in parameters of the scotopic sensitivity function (e.g. Vmax of the Naka Rushton fit *p* = 0.03) were observed in non-treated *vs.* ketamine-xylazine treated animals.

**Conclusions:**

Our results suggest that pre-treatment with ketamine-xylazine anesthesia protects retinas against light damage, reducing photoreceptor cell death. These data support the notion that anesthesia with ketamine-xylazine provides neuroprotective effects in light-induced cell damage.

## Introduction

The term neuroprotection refers to an intervention that may prevent, retard or reverse neuronal cell death after acute or during chronic insult [1]. The general aim of neuroprotective strategies is to minimize damage and/or maximize recovery by influencing underlying etiology or pathogenesis [1], [2]. Evidence of the neuroprotective effect of anesthetics includes the capacity of general anesthesia to increase neuronal tolerance to hypoxic and ischemic insults [3], [4] and their protective effects in neurodegenerative diseases [5] including Alzheimer [6] and animal models of Parkinson [7]. However, limited evidence is available on the effects of anesthetic agents in terms of retinal neuroprotection. In axotomized rat retinal ganglion cells (RGC) neuroprotection has been reported *in vivo* when combinations of chloral hydrate-buprenorphine, ketamine-xylazine or fentanyl-medetomidine-midazolam were used for anesthesia; chloral hydrate alone or in combination with carprofen did not affect the numbers of surviving RGCs [8].

The ability of light to cause severe retinal degeneration has been well described [9], [10]. Retinal light damage is indeed used as model for human retinal degeneration (RD) arising from environmental insult, aging and genetic disease [11]. However, published protocols for light damage differ in exposure time, illumination strength and emission spectrum of the applied light source [10]. Here, we used a very similar protocol as described by Grimm et al. to induce profound retinal degeneration [12].

In a model of light-induced RD, halothane, a gaseous inhalation anesthetic agent, led to protection against photoreceptor apoptosis [13]. Another anesthetic regimen, ketamine-xylazine, is widely used in experimental research [14], and is recommended in studies of the central and peripheral nervous system [15], [16]. However, there are as of yet no reports on the effect of this commonly used anesthetic regimen on RD, even though many studies in murine models of induced and/or hereditary RD use ketamine-xylazine for narcosis in *in vivo* analyses to determine the natural disease progression or the effect of experimental therapeutic interventions on the course of RD. Given the above mentioned evidence in the literature on the potential neuroprotective influence of anesthetics, it is of considerable interest to analyze whether ketamine-xylazine might have potential confounding effects on RD. Therefore, our study aimed to determine the effect of ketamine-xylazine anesthesia in a light-induced model of RD in rats.

## Methods

### Ethic statement

All procedures were performed in compliance to the Statement of the Association for Research in Vision and Ophthalmology Statement for the use of animals in Ophthalmic and Visual Research and approved by the Tuebingen University committee on animal protection. Protocols compliant the German law on animal protection were reviewed and approved by the Regional Council in Tuebingen (approval ID AK 10/09). All efforts were made to minimize the number of animals used and their suffering.

### Animals

Twenty-seven Sprague Dawley® rats (Charles River Laboratories GmbH, Sulzfeld, Germany) with a mean weight of 218.32 g±21.40 g (mean ± standard deviation (SD)) at baseline were included. The rats were housed under standard laboratory conditions with light-dark cycles of 12 h/12 h under room illuminations of 200 lux.

### Anesthesia and Light Exposure

Before experimental light exposure animals were dark adapted for 12 h. All preparatory steps prior to light exposure were performed under dim red light. Three experimental groups were considered (Table 1). The light damage (LD) group was exposed to light without prior anesthesia. The group with light damage and prior anesthesia (LDA) was kept under anesthesia for 1 h with a combination of ketamine 100 mg/kg and xylazine 5 mg/kg (WDT eG, Garbsen, Germany) injected intraperitoneally and kept in darkness after anesthesia for 2 h to awake, before light exposure. All rats were awake before light damage. The pupils were dilated using one drop of tropicamide (Mydriatikum® 0.5%, Stulln, Germany) 30 minutes before light damage. The cages were lined with aluminum foil to reach an environmental illumination. During light exposure, the temperature in the cages and the rats were checked every 15 minutes to avoid sleeping or eye closure. The LD and LDA groups received light exposure for 2 h under a mean brightness of 16 000 lux (4 Philips TLD 965® lamps, Hamburg, Germany) and subsequently were again kept in darkness for 12 h [17]. A third group was not anesthetized or illuminated (control).

**Table 1:** Experimental groups used in this study.

*Group*	*Abbreviation*	*exposed to light*	*ketamine-xylazine anesthesia*	*measurement*
Light damage with prior anesthesia	LDA	yes	yes	ERG, *in vivo* imaging and histology
Light damage only	LD	yes	no	ERG, *in vivo* imaging and histology
Control	-	no	no	*In vivo* imaging and histology

### Electroretinography (ERG)

Baseline ERG was recorded in all rats of the LD and LDA group at the beginning of the study prior to light exposure for intra-individual comparison at follow-up examinations, which were performed once a week for 2 consecutive weeks after light damage. The ERG was measured after a period of 12 h of dark adaptation. To dilate the pupils one drop of tropicamide (Mydriatikum® 0.5%, Stulln, Germany) was applied 20 minutes before the ERG measurement started. A Dawson-Trick-Litzkow electrode was used as active electrode [18]. Methocel® eye-gel (2% methylcellulose, Omnivision GmbH, Puchheim, Germany) was applied to avoid exposure keratopathy. Two subcutaneous needle-electrodes (Ambu®Neuroline Twisted Pair Subdermal, Bad Nauheim, Germany) one inserted between the eyes and the other in the tail served as reference and ground electrodes, respectively. An acceptable impedance level of <10 k*Ω* at 25 Hz was ensured before and during the ERG recording.

The dark adapted ERG protocol consisted of 16 steps with increasing stimulus strength from 3×10^−5^ to 60 scot cd.s/m^2^, which were produced by a mixed light (white 6500 K) using a Ganzfeld stimulator (ColorDome®, Diagnosys LLC, Cambridge, GB). The duration of flashes was 4 ms. All scotopic flashes were delivered without background illumination and constant inter-stimulus-intervals of 1 s for dim flashes and up to 45 s for bright flashes to ensure stable dark adapted conditions. Band-pass filtering was applied from 0.3 to 300 Hz using the machine's built-in software algorithm (Espion®, Diagnosys LLC, Cambridge, GB). Averages ranged from 20 sweeps for dim flashes to 2 sweeps for bright flashes. The photopic ERG protocol consisted of an initial light adaptation phase with a background illumination of 30 cd/m^2^ (white 6500 K) for 10 minutes. For single flash responses five steps with increasing stimulus strengths from 0.3 to 20 phot cd.s/m^2^ were chosen. Three flicker steps from 6 to 20 Hz were applied with constant stimulus strength of 3 cd.s/m^2^. Twenty sweeps were averaged for single flash responses and 30 sweeps for flicker stimulation.

### *In vivo* retinal imaging

Imaging was performed in all rats of the LD and LDA group 36 h and 7 d after light exposure. Data from control animals served as reference. For confocal scanning laser ophthalmoscopy (cSLO) and spectral domain optical coherence tomography (SD-OCT) imaging a Spectralis™ HRA+OCT device (Heidelberg Engineering, Germany) was used as previously described [19]. The near-infrared channel at 795 nm was used for en face cSLO imaging of the peripapillary central region. Vertical and horizontal SD-OCT cross-sections centered on the optic disc were recorded consecutively to ensure perpendicular orientation of peripapillary retinal tissue and thereby avoiding oblique recordings. Quantification of central retinal thickness based on high resolution vertical line scans was performed using the proprietary software from Heidelberg Engineering (Eye Explorer version 1.6.4.0, HRA/Spectralis Viewing Module version 5.3.2.0). Briefly, total retinal thickness (TRT) and outer nuclear layer thickness (ONL) were quantified at eight equidistant loci, 250 µm apart, towards the periphery along a vertical meridian (superior and inferior central, mid-central, mid-peripheral and peripheral) in LDA, LD and control animals. For this, segmentation lines were manually placed to detect TRT (vertical distance from inner limiting membrane to retinal pigment epithelium) or ONL (vertical distance between outer plexiform layer and outer limiting membrane), respectively. Each line scan covered a distance of ca. 2 mm.

### Histology

The rats were sacrificed with CO_2_, the eyes immediately enucleated and the anterior parts and lenses removed; for paraffin sectioning, fixed eyecups (4% paraformaldehyde (PFA) in 0.1 M phosphate buffer (PB; pH 7.4) for 1 h at 4°C) were dehydrated in EtOH, immersed in Chloroform and embedded in paraffin. Radial 5 µm sections were stored at 4°C.

Histologic methods included the quantification of outer nuclear layer (ONL) thickness and inner/outer segments (IS/OS) length, which was measured at four equidistant positions, ∼300 µm apart, starting next to the optic nerve along the superior and inferior hemiretina.

### Immunohistochemistry

Tissue sections were deparaffinized and rehydrated. Antigen retrieval was achieved by pressure cooking in 0.1 M citrate buffer, pH 6 for 10 minutes, followed by cooling at room temperature before incubation with the antibodies. Radial sections were preincubated with phosphate buffered saline (PBS; 50 mM, pH 7.4) containing 20% normal goat serum and 0.03% Triton X-100 (Sigma-Aldrich) for 2 hours at room temperature in order to block nonspecific antibody binding. Subsequently, the sections were incubated overnight at 4°C with specific primary antibodies. The following antibodies were used: Rhodopsin clone RET-P1 (Mouse, mAb, 1∶400, Millipore Chemicon) and Glial Fibrillary Acidic Protein (GFAP) Clone G-A-5 (Mouse, mAb, 1∶400, Sigma).The immunoreaction was visualized with Alexa Fluor 488 anti-rabbit antibody (Rockland, Gilbertsville, PA) diluted 1∶750. Controls were carried out by omitting the first antibody. All micrographs were taken from the superior region of the retina using an Olympus AX70 microscope. Images shown in figures are representative for least three different animals for each group.

### TUNEL Assay

Terminal deoxynucleotidyltransferase-mediated biotinylated UTP nick end labeling (TUNEL) staining [20] was performed using an in situ cell death detection kit (Fluorescein or TMR; Roche Diagnostics GmbH, Mannheim, Germany) as suggested by the manufacturer.

### Data analysis

ERG data extraction and analysis was performed using previously described software [21], [22]. B-waves were analyzed after extracting oscillatory potentials using a discrete Fourier transform algorithm with a low and high cut-off of 75–300 Hz. For low intensity flashes an estimate of rod sensitivity was fitted using the Naka-Rushton paradigm [23]. ERG responses of 9 steps (0.000003–0.03 scot cd.s/m^2^) were related to stimulus strength (scot cd.s/m^2^) and parameters of the Naka-Rushton fit (*V_max_* as maximum response; *k* as stimulus strength needed for 50% of *V_max_*; *n* as gradient of fit) were extracted (Formula (1)).
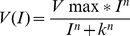
(1)


A-wave and b-wave amplitudes and implicit times of the mixed rod-cone responses (0.3–60 scot cd.s/ms), the photopic single flash and the flicker responses (only b-waves) were analyzed. Additionally, a-wave slopes were compared between groups. Oscillatory potentials (OP) were analyzed after offline band-pass filtering (75 to 300 Hz) of responses elicited by intense scotopic flash stimuli (0.3–60 scot cd.s/m^2^). OPs were automatically calculated as area-under-the-curve (AUC) by the software between the a-wave and the peak of the b-wave.

### Statistical Analysis and cell counting

Statistical analyses were performed using JMP® software (version 8.0.2, SAS Institute Inc., Cary, NC, USA). ERG parameters were compared using intra-individual ratios between baseline and follow-up examination with analysis of variances (ANOVA) and Tukey's post hoc test. All OCT and histological results are expressed as mean ± standard deviation (SD) from at least three animals in each group and significance was tested using unpaired, two-tailed Students t-test. Significance level was set at <0.05. Histological and OCT data included measurements of healthy control rats for descriptive comparison. This group was measured only once and not used for statistical analysis. For cell quantifications, pictures of whole radial slices were captured using Mosaic mode of AxioVision**™** 4.7 at 20× magnification. Labelled cells were counted manually. The total number of cells was determined by dividing outer nuclear layer (ONL) area through average cell size. The number of positive cells was then divided by the total number of ONL cells giving the percentage of positive cells.

## Results

### ERG

**Figure 1:**
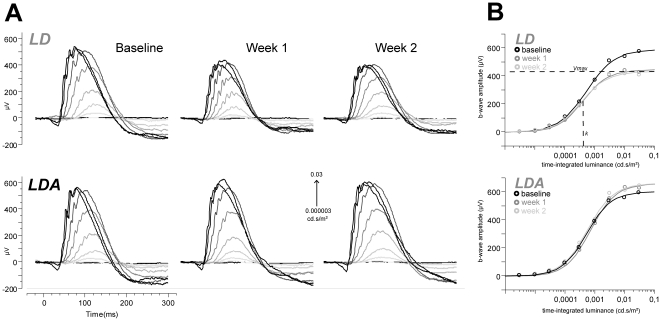
Scotopic sensitivity function and corresponding ERG curves in LD and LDA rats. (A) ERG responses to 9 flashes of increasing stimulus strength (0.000003–0.03 cd.s/m^2^) under dark-adapted condition for representative rats of each group (LD, LDA). Examinations are shown for baseline measurement, 7 days (7 d) and 14 days (14 d) after light damage. A decrease of ERG potentials were indicated in the LD group after light damage. The LDA group shows an increase of amplitudes after light damage due to normal growth. (B) Scotopic fits (described by *Vmax*, *k* and *n*) of the same measurements as illustrated in A: The b-wave amplitudes (µV) were fitted against stimulus strength (cd.s/m^2^). Points demonstrate single ERG b-waves with increasing stimulus strength. The fits show the increase in amplitudes during the whole study period in the LDA group and the decrease of amplitudes in the LD group after light damage (note the difference between baseline and 7 d and 14 d in both groups).

To assess functional properties and potential differences between LD and LDA retinas after light damage (Table 1) we analyzed ERG responses. No statistically significant differences were found between groups at baseline measurements. Following light damage the scotopic sensitivity function, described by a Naka-Rushton fit, yielded significant differences in the maximum response (*V_max_*) (Fig. 1) between LD and LDA groups (ANOVA *p* = 0.05 at day 7 and *p* = 0.03 at day 14 after light damage) with higher amplitudes after light damage for the LDA group, indicating a positive influence on function and/or remaining number of rod on-bipolar cells and on Müller cells. k (as a value for the stimulus strength needed to evoke a half-maximal response or 50% of *V_max_*) remained unchanged during the study (ANOVA *p* = 0.67 at 7 d and *p* = 0.68 at 14 d), indicating that remaining cells functioned properly. This finding suggests that retinal cells of the rod system are reduced in quantity, but remaining cells are functionally unaffected.

**Figure 2:**
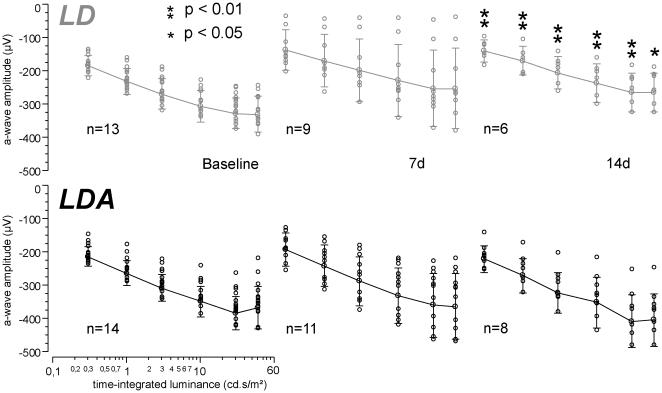
Comparison of a-wave amplitudes between LD and LDA rats at rising stimulation strengths. A-wave analysis at different stimulus strengths (0.3–60 cd.s/m^2^) measured under dark adapted condition. For descriptive analysis means were connected (line) and standard deviations were marked (whiskers). Single measurements of a-waves are illustrated by points. A significant difference between both groups (LD vs. LDA) was detected at 14 days after light damage (* = *p*<0.05, ** = *p*<0.01). Higher amplitudes were measured for the LDA group.

For high intensity stimuli (0.3–60 cd.s/m^2^) under scotopic conditions comparison of a-waves as indicator for the photoreceptor function of rods and cones showed higher amplitudes for the LDA group in comparison to the LD group (Fig. 2) at all time points, reaching significance only at 14 d (ANOVA *p* = 0.01 at 3 cd.s/m^2^). Implicit times of a-waves did not differ between groups at any time point. This indicates preservation of photoreceptor function in the LDA group. The a-wave slopes also reached significantly higher values for the LDA group 14 d after light exposure (ANOVA *p* = 0.01 for 3 cd.s/m^2^). Since the a-wave slope can be regarded as electrophysiological indicator of the phototransduction process in the photoreceptors, this finding suggests a preservation of the phototransduction and hence of the photoreceptors in the LDA group.

**Figure 3:**
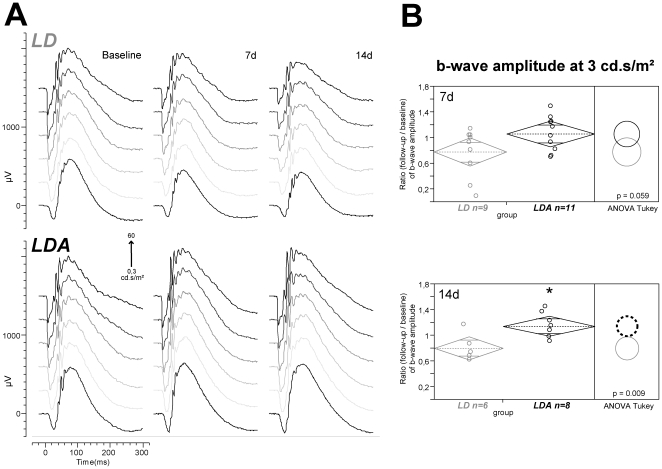
Mixed rod-cone b-wave amplitudes compared between LD and LDA rats. (A) Examples of ERG waves elicited by stimulus strengths rising from 0.3 to 60 cd.s/m^2^ under dark adapted conditions for both groups (LD, LDA) and three measurements (baseline, 7 days (7 d) and 14 days (14 d) after light damage). A decrease of amplitudes was detected after light damage in the LD group. The application of anesthesia before light damage (LDA group) could protect retinal function against degeneration. (B) Statistical analysis of b-wave amplitudes at 3 cd.s/m^2^ under dark adapted conditions illustrated by diamonds of agreement with mean (middle line within the diamonds) and 95% confidence interval (upper and lower corners of the diamonds). The dashed circle indicates the statistical significant difference between groups (LDA with significant higher amplitudes marked black [group and circle]; ANOVA with post hoc Tukey's analysis *p* = 0.059 at 7 d and *p* = 0.009 at 14 d).

Intra-individual ratios of b-wave amplitudes at high intensity stimuli (0.3–60 cd.s/m^2^; Fig. 3A) showed higher potentials after light damage for the LDA group during the follow-up examinations in comparison to the LD group (ANOVA p = 0.009 at 3 cd.s/m^2^ and 14 d; Fig. 3B) and unchanged implicit times. At this intensity range, mixed b-wave responses mainly originate from rod and cone bipolar cells and Müller glia cells. As such, functional preservation points to a structural rescue of these cell populations in the LDA compared to the LD group.

Analysis of photopic responses (flicker and single flash) and the OPs revealed no differences between both groups (data not shown). Thus, no functional influences were observed on isolated cone function.

### cSLO and OCT

**Figure 4:**
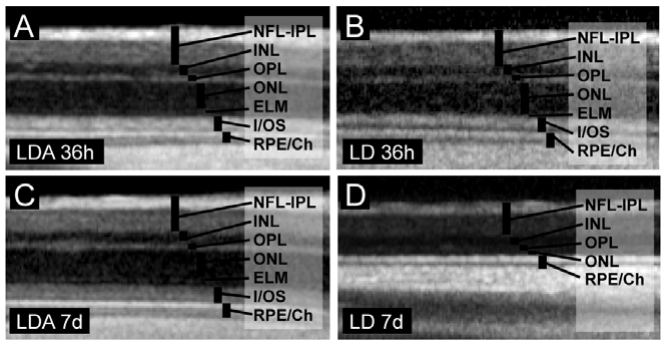
Representative virtual cross sections from noninvasive SD-OCT imaging in LDA and LD retinas after light exposure. 36 h (upper panel) and 7 d (lower panel). (A, C) LDA cross sections maintained similar laminar architecture at both time points. Signal composition in (B) LD retina 36 h after light exposure appeared similar compared to (A) LDA retina at the same time point. However, (D) 7 days after light exposure LD retina revealed near complete loss of ONL and photoreceptor inner/outer segments. LD: Animals with light exposure; LDA: Animals with ketamine-xylazine anesthesia before light exposure; NFL-IPL: Nerve fiber layer - inner plexiform layer; INL: Inner nuclear layer; OPL: Outer plexiform layer; ONL: Outer nuclear layer; ELM: External limiting membrane; I/OS: Inner/outer segment border; RPE/Ch: Retinal pigment epithelium/Choroid.

**Figure 5:**
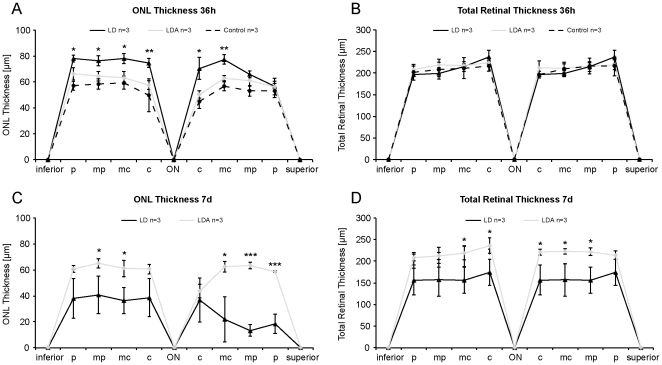
In vivo analysis of ONL and total retinal thickness in LD (n = 3), LDA (n = 3) and control retinas (n = 3). 36 h (A, B) and 7 d (C, D) after light exposure. Measurements were taken at equidistant positions (c = central, midcentral = mc, midperipheral = mp and inferior/superior periphery = p) on virtual cross sections centered on the optic nerve (ON). All data are reported as mean ± standard deviation (whiskers). (A) 36 h after light exposure, animals in the LD group showed increased ONL thickness compared to LDA and control animals. (B) However, total retinal thickness remained unchanged at this early time point. (C) One week after light exposure, LD animals showed a dramatic loss of ONL thickness, while LDA animals remained at control levels of ONL thickness. (D) Retinal thinning in the LD group was now also evident in the total retinal thickness. LD: Animals with light exposure; LDA: Animals with ketamine-xylazine anesthesia before light exposure; Control: No anesthesia or light exposure. (* = *p*<0.05, ** = *p*<0.01, *** = *p*<0.001).

*In vivo* cSLO and OCT imaging of the retina was performed to detect structural changes in vivo such as edema formation or neuronal degeneration [19], [24]. Imaging data at the first time point, 36 h post light damage (Fig. 4A–B, 5A–B), did not reveal structural changes with normal retinal layering (e.g. outer nuclear layer, ONL) and physiological total retinal thickness (TRT) in the LDA group (TRT_LDA_ = 216.13 µm±9.36 SD, ONL_LDA_ = 60.29 µm±6.13 SD) compared to control animals (TRT_control_ = 209.67 µm±16.85 SD, ONL_control_ = 54.04 µm±6.92 SD). Conversely, LD animals demonstrated a significant increase in ONL thickness (ONL_LD_ = 72.17 µm±8.14 SD) compared to LDA (*p*<0.01) at this early time-point, while no significant increase was found at the level of total retinal thickness (TRT_LD_ = 212.08 µm±18.72 SD; *p* = 0.35). However, one week after light damage (Fig. 4C–D, 5C–D), both total retinal thickness and outer nuclear layer thickness were significantly reduced in the LD *vs.* the LDA group (TRT_LD_ = 161.08 µm±29.04 SD vs. TRT_LDA_ = 219.38 µm±12.24 SD, *p*_TRT_<0.01 and ONL_LD_ = 30.50 µm±15.18 SD vs. ONL_LDA_ = 59.42 µm±7.14 SD, *p*_ONL_<0.01). *In vivo* imaging data thus suggests retinal edema formation 36 h after light damage and subsequent loss of total and more specifically ONL thickness, which would be expected in photoreceptor cell death [19], [24]. The complete set of data is available as supplemental data (Table S1).

### Histology

#### 36 h after light-induced damage

**Figure 6:**
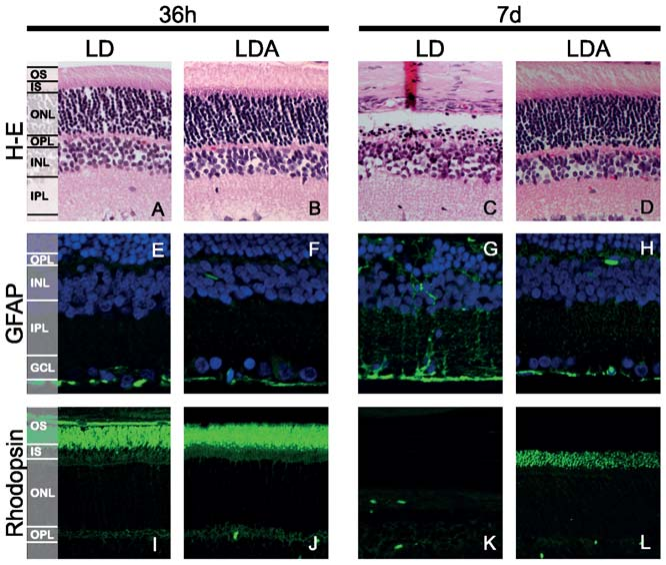
Retinal sections from LD and LDA retinas. 36 h (left panel) and 7 d (right panel) after light exposure. Hematoxylin and eosin staining (A–D) GFAP staining (E–H) and rhodopsin staining (I–L). 7 d after light exposure, LD retinas showed an important decrease in the photoreceptor cell numbers and disappearance of IS/OS, while morphological degenerative changes in the INL and GFAP expression on Müller cells indicating gliosis became apparent. Ketamine-xylazine anesthesia before light exposure preserved retina morphology, increased photoreceptor survival and prevented gliosis LD: Animals with light exposure; LDA: Animals with ketamine-xylazine anesthesia before light exposure. GCL: ganglion cell layer; IPL: inner plexiform layer; INL: inner nuclear layer; OPL: outer plexiform layer; ONL: outer nuclear layer; IS/OS: inner/outer segments.

**Figure 7:**
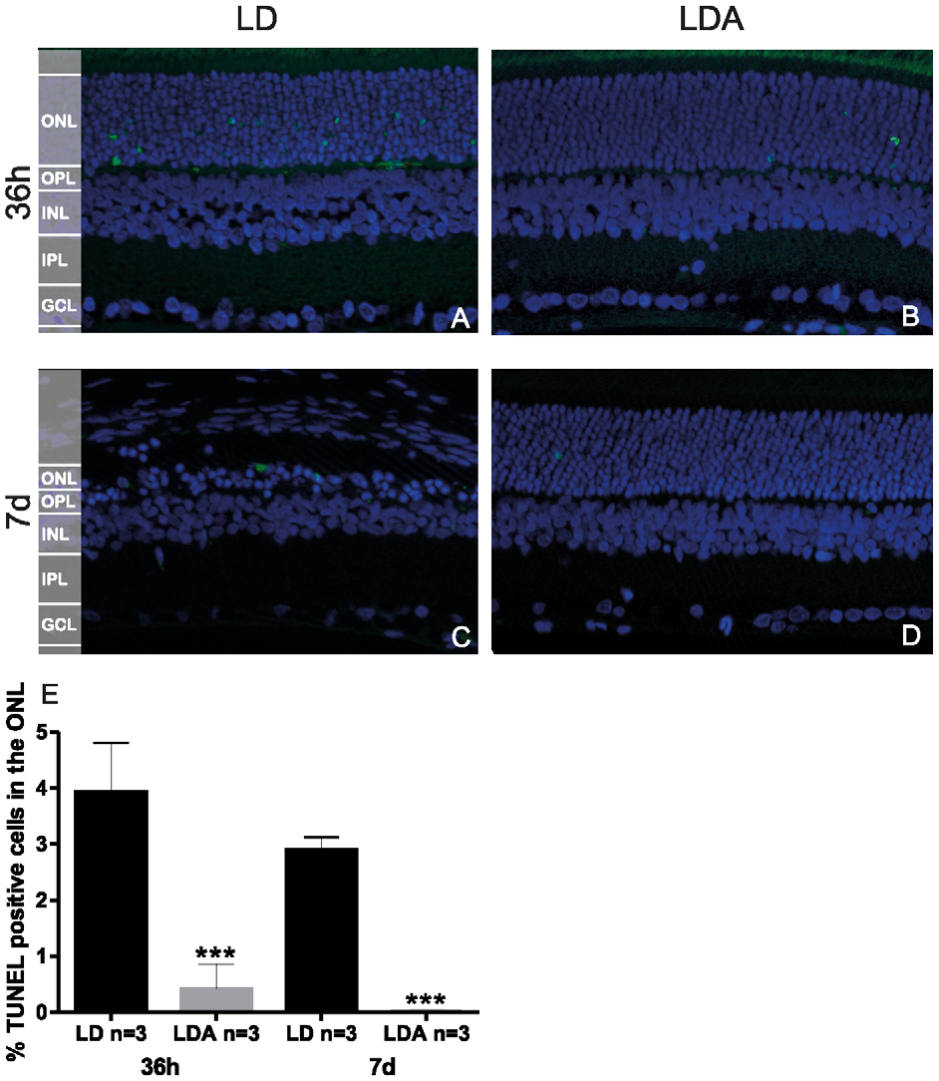
TUNEL staining and quantification of photoreceptor cell death in LD and LDA retinas. 36 h (A and B) and 7 d (C and D) after light exposure. At both points, significant protection was seen with ketamine-xylazine anesthesia. (*** = *p*<0.001). Mean + SD, n = 3. LD: Animals with light exposure; LDA: Animals with ketamine-xylazine anesthesia before light exposure. GCL: ganglion cell layer; IPL: inner plexiform layer; INL: inner nuclear layer; OPL: outer plexiform layer; ONL: outer nuclear layer.

**Figure 8:**
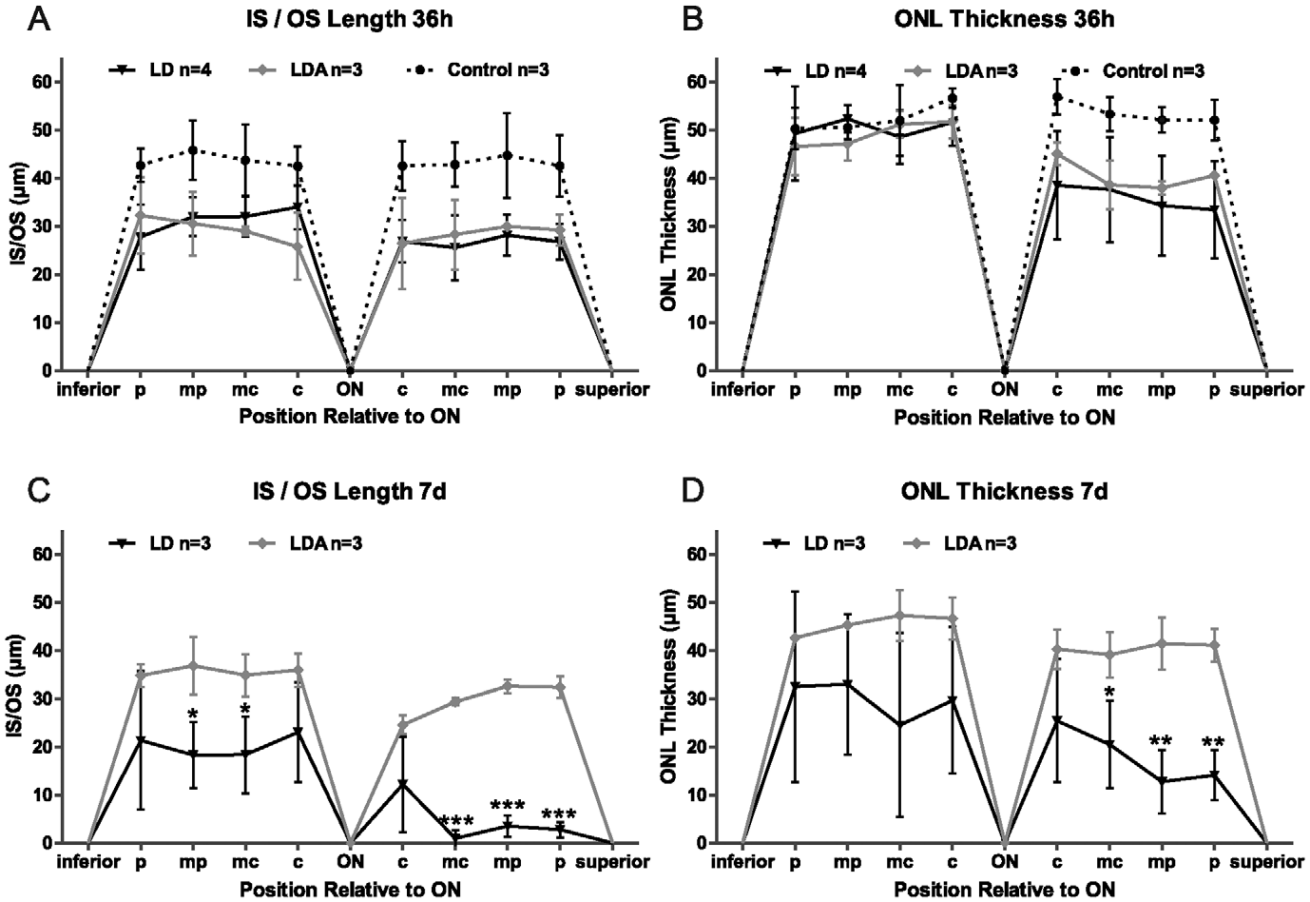
Comparison of photoreceptors IS/OS length and ONL thickness between LD, LDA and control retinas. Measurements on histological retinal sections 36 h (A, B) and 7 d (C, D) after light exposure. The equidistant positions (c, mc, mp and p, inferior and superior) of retinal sections were analyzed around the optic nerve (ON). Measurements are shown as mean (point) and standard deviation (whiskers). 36 h after light exposure, animals showed a reduction in the IS/OS length, in inferior and superior retina (B) and a decrease of ONL thickness was observed only in the superior retina in both LD and LDA. (B). 7 d after light exposure, an important decrease in the IS/OS length and in the ONL thickness were found in LD group when compare to LDA group in the superior retina. (n = 3) LD: Animals with light exposure; LDA: Animals with ketamine-xylazine anesthesia before light exposure; Control: No anesthesia or light exposure. (* = *p*<0.05, ** = *p*<0.01, *** = *p*<0.001).

Representative sections from the superior retina are shown in Figure 6A and B. No difference was found regarding GFAP staining (Fig. 6E and F) or Rhodopsin (Fig. 6I and J) immunolabelling between LDA and LD groups. Rhodopsin staining showed a normal distribution and was restricted to photoreceptor OS. We performed TUNEL assay to detect cell death [20]. At this early time point, LD retinas demonstrated higher levels of TUNEL positive cells in the ONL when compared to LDA (LD: 3.94%±0.71 SD, n = 3; LDA: 0.42%±0.36 SD, n = 3, *p<*0.003) (Fig. 7A, B and E). In control retinas without induced light damage, no TUNEL-positive cells were observed (data not shown). Exposure to high levels of light induces shortening of photoreceptor inner and outer segments (IS/OS) (Fig. 8A) in both, the LD and the LDA groups compared to control retinas. No differences of IS/OS length were observed between inferior and superior retina. Both groups showed a strong reduction of the ONL thickness restricted to the superior retina; ONL thickness in the inferior retina was normal (Fig. 8B). Interestingly, increased ONL thickness as observed in OCT imaging of LD retinas (Fig. 4 and 5) was not found in the ex vivo histological observation. This might be due to tissue processing (e.g. dehydration and fixation), which would be expected to obscure e.g. retinal edema formation [19].

#### 7 d after light-induced damage

Inferior to superior cross-sections through the retina prepared 7 days after light exposure revealed a pronounced loss of photoreceptors in the LD group when compared to the LDA group (6C and D) and demonstrated a statistically significant reduction of the ONL thickness in the superior retina (Fig. 8D, *p<*0.01). This result matches our OCT observations (Fig. 5C). The LD group showed a clear reduction in the IS/OS length in the remaining photoreceptor cells, predominantly in the superior retina (Fig. 8C, *p<*0.001). Reduction of OS was confirmed by absence of rhodopsin immunolabelling (Fig. 6 L). In the LD group GFAP immunoreactivity was up-regulated indicating Müller cell gliosis (Fig. 6H). At this later time point the number of TUNEL positive cells decreased in both groups, however, the percentage of stained cells in the ONL was still significantly increased in the LD group compared to the LDA retinas (LD: 2,89%±0.19 SD, n = 3; LDA: 0.02%±0.02 SD, n = 3, *p<*0.001) (Fig. 7C–E). The complete set of data is available as supplemental data (Table S2).

## Discussion

Here we present novel evidence that anesthesia with ketamine-xylazine has a protective effect on photoreceptor cell death caused by light exposure in rats. Several trials indicate neuroprotective effects of anaesthetic drugs on the CNS [25] with various degrees of neuroprotection being observed when different paradigms of intravenous, inhaled or combined anesthesia are compared (for a summary of the current literature of the most commonly used anaesthetic drugs and their role in neuroprotection see Schifilliti et al 2010) [5]. Protective effects of anesthetics, specifically on the retina, have also been described before [5], [8], [10], [13] and several explanations for its mechanism were suggested. However, the great diversity of protocols e.g. in light-induced retinal degeneration experiments makes it difficult to conclusively interpret these results. Numerous anesthetics and administration procedures, different ages and animals used, diverse parameters to measure the retinal damage and different techniques used to induce the light damage are some examples [8], [13], [26]. Several studies point to a neuroprotective effect of intravenous or volatile anesthetics [5] also on axotomized retina [8] and animal models with light-induced retinal degeneration [10], [13].

Anesthetics like halothane can reversibly inhibit metabolic rhodopsin regeneration and thus prevent rhodopsin from absorbing high numbers of photons during light exposure [13]. In their study, Keller et al. found that anesthesia with halothane prevented retinal degeneration induced by white light, but not by blue light [13]. Similar results were observed by Grimm et al. (2001) who concluded that damage related with blue light was rhodopsin mediated [26]. Another study confirmed that light damage only occurred when the retina was supplied with 11-cis retinal, the chromophore of rod and cone opsins. These data suggested that it was indeed the halothane mediated block of regenerated 11-cis retinal reincorporation that conveyed the neuroprotective effect against light damage [10].

The quality of light used to induce retinal degeneration, its exposure duration and intensity determine the severity of the degeneration in light-induced damage [10]. Our light exposure paradigm with an emission spectrum peaking in the blue range results in rapid retinal degeneration, comparable with the damage observed after short-term exposure to blue light [13], [26]. Similar to the effects observed under halothane anesthesia [13], in our study, a single injection of ketamine-xylazine before light exposure was enough to prevent light-induced damage.

Because ketamine is a poor muscle relaxant [14] it is commonly used in combination with other anesthetics such as xylazine. Xylazine is an alpha_2_ adrenergic agonist, similar to clonidine and produces its effects by interaction with central and peripheral alpha_2_adrenoreceptors, and it is used in veterinary medicine as a sedative, analgesic and muscle relaxant [27]–[29]. However, neuroprotective effects of ketamine have been seen with or without using xylazine, suggesting that the neuroprotective effects might be related with the use of ketamine [28].

While ketamine-xylazine has been shown to slow but not block metabolic rhodopsin regeneration in rats [13], there are other mechanisms that have been suggested to explain protection against light-induced damage. These include activation of nitric oxide synthase, increased intracellular calcium, generation of oxidative stress and disturbed mitochondrial function [10], [30]. Interestingly, it has been shown that ketamine increases neuronal and astroglial viability, preserves neuronal morphology, reduces cell swelling after anoxia-hypoxia or glutamate injury, preserves cellular energy status after ischemic insults and preserves ATP production [5], [31]. Ketamine is a non-competitive antagonist of NMDA receptors and is known to inhibit the transcriptional activity of the nuclear factor kappa Beta (NF-kappaB) in CNS [5], [32], [33]. NF-kappaB is activated in many neurodegenerative diseases and also in the inherited retinal degeneration model, *rd* mice [34] and light-induced retinal degeneration in mice [35].

In line with previous reports [36], [37], we have found a localized region in the superior retina of LDA and LD rats that shows particularly light-induced outer segment shortening and photoreceptor loss. The reason for preferential damage in this sensitive region is not completely understood but may be related to the greater rhodopsin levels and ROS length in the superior hemisphere of the rat eye [38].

The primary purpose of this study was to investigate, whether the protocol for anesthesia using ketamine-xylazine, which is very common in vision science and beyond, demonstrates a neuroprotective effect in the light damage paradigm. The results of this study clearly show a strong and potentially confounding effect, which previously had remained undetected. This finding has potential implications on the interpretation of existing literature and future experimental designs using light damage to induce retinal degeneration in animals. However, the cross sectional design and relatively small number of animals in the morphological assessments (cSLO/SD-OCT, Histology) warrant further studies to assess the biologic relevance of the neuroprotective effect. Further studies are needed to dissect the mechanism of ketamine-xylazine neuroprotection and analyze, whether it also affects other forms of retinal degeneration. It would also be interesting to compare the mechanism of halothane vs. ketamine-xylazine, including measurement of rhodopsin levels during light damage exposure and also pre-treatment with each drug of the combination by itself.

In summary, our data indicate that pre-treatment with ketamine-xylazine anesthesia protects retinas against light-induced damage, reducing photoreceptor cell death. Consequently, the neuroprotective properties of this widely used anesthetic mixture must be taken into account when interpreting results of studies on neuroprotection in these animals.

## Supporting Information

Table S1 **In vivo ONL thickness data from superior and inferior retinal locations in light damage only (LD) and light damage with prior anesthesia (LDA) groups.**
(DOCX)

Table S2 **ONL thickness data from corresponding fields from the superior and inferior retina in light damage only (LD) and light damage with prior anesthesia (LDA) groups.**
(DOCX)
