# Correction: Effects of Combined Ketamine/Xylazine Anesthesia on Light Induced Retinal Degeneration in Rats

**DOI:** 10.1371/annotation/d52a610e-6a56-4158-90bf-45e37f053567

**Published:** 2012-08-06

**Authors:** Blanca Arango-Gonzalez, Andreas Schatz, Sylvia Bolz, Javier Eslava-Schmalbach, Gabriel Willmann, Ahmad Zhour, Eberhart Zrenner, M. Dominik Fischer, Florian Gekeler

The following reference was omitted from the article:

Wen R, Cheng T, Li Y, Cao W, Steinberg RH. Alpha 2-adrenergic agonists induce basic fibroblast growth factor expression in photoreceptors in vivo and ameliorate light damage. J Neurosci. 1996 Oct 1;16(19):5986-92.

Our findings were supported by the work of Wen et al. who showed a protection of light induced photoreceptor degeneration after multiple injections of xylazine (4 to 7 injections). The protective mechanism by xylazine, an a2-agonist has been traced back to the expression of bFGF. However these findings were shown for a different light damage paradigm (7 days of light exposure) compared to the one used in this present study (single exposure for 2h). Additionally, our study investigated the effect of a single application, rather than multiple injections, of xylazine/ketamine, thus mimicking the widely used paradigm for procedural anesthesia in rodents.

