# Supplementary material for: Effects of Combined Ketamine/Xylazine Anesthesia on Light Induced Retinal Degeneration in Rats
Source: PLoS One. 2012 Apr 25;7(4):e35687. doi: 10.1371/journal.pone.0035687 (PMC3338443; doi:10.1371/journal.pone.0035687)
Supplement: Table S1 — In vivo ONL thickness data from superior and inferior retinal locations in light damage only (LD) and light damage with prior anesthesia (LDA) groups. (DOCX) [file pone.0035687.s001.docx]

**Table S1.** In vivo ONL thickness data from superior and inferior retinal locations in light damage only (LD) and light damage with prior anesthesia (LDA) groups.

| Position Relative to ON | | 36h | | | | | | | | | 7d | | | | | | | | |
| --- | --- | --- | --- | --- | --- | --- | --- | --- | --- | --- | --- | --- | --- | --- | --- | --- | --- | --- | --- |
|  |  | LD n=3 | | | Mean ± SD | LDA n=3 | | | Mean ± SD | *p* | LD n=3 | | | Mean ± SD | LDA n=3 | | | Mean ± SD | *p* |
| Inferior | p | 76 | 81 | 77 | 78.00±2.65 | 64 | 63 | 72 | 66.33±4.93 | 0.0226 | 42 | 21 | 51 | 38.00±15.39 | 58 | 61 | 63 | 60.67±2.52 | 0.0656 |
|  | mp | 79 | 72 | 78 | 76.33±3.79 | 61 | 64 | 68 | 64.33±3.51 | 0.0158 | 35 | 30 | 57 | 40.67±14.36 | 66 | 61 | 68 | 65.00±3.61 | 0.0466 |
|  | mc | 74 | 82 | 78 | 78.00±4.00 | 69 | 61 | 60 | 63.33±4.93 | 0.0161 | 30 | 31 | 48 | 36.33±10.12 | 68 | 56 | 59 | 61.00±6.24 | 0.0229 |
|  | c | 75 | 71 | 78 | 74.67±3.51 | 54 | 61 | 58 | 57.67±3.51 | 0.0041 | 37 | 25 | 54 | 38.67±14.57 | 59 | 65 | 58 | 60.67±3.79 | 0.0646 |
| Superior | c | 78 | 72 | 61 | 70.33±8.62 | 47 | 52 | 52 | 50.33±2.89 | 0.0189 | 36 | 20 | 54 | 36.67±17.01 | 45 | 48 | 38 | 43.67±5.13 | 0.5325 |
|  | mc | 79 | 80 | 73 | 77.33±3.79 | 65 | 61 | 63 | 63.00±2.00 | 0.0044 | 10 | 14 | 42 | 22.00±17.44 | 63 | 66 | 58 | 62.33±4.04 | 0.0175 |
|  | mp | 68 | 63 | 67 | 66.00±2.65 | 63 | 56 | 64 | 61.00±4.36 | 0.1647 | 16 | 16 | 8 | 13.33±4.62 | 61 | 66 | 63 | 63.33±2.52 | 0.0001 |
|  | p | 52 | 59 | 59 | 56.67±4.04 | 60 | 49 | 60 | 56.33±6.35 | 0.9425 | 18 | 26 | 11 | 18.33±7.51 | 59 | 59 | 58 | 58.67±0.58 | 0.0007 |
