# Supplementary material for: Effects of Combined Ketamine/Xylazine Anesthesia on Light Induced Retinal Degeneration in Rats
Source: PLoS One. 2012 Apr 25;7(4):e35687. doi: 10.1371/journal.pone.0035687 (PMC3338443; doi:10.1371/journal.pone.0035687)
Supplement: Table S2 — ONL thickness data from corresponding fields from the superior and inferior retina in light damage only (LD) and light damage with prior anesthesia (LDA) groups. (DOCX) [file pone.0035687.s002.docx]

**Table S2.** ONL thickness data from corresponding fields from the superior and inferior retina in light damage only (LD) and light damage with prior anesthesia (LDA) groups.

| Position Relative to ON | | 36h | | | | | | | | | | 7d | | | | | | | | |
| --- | --- | --- | --- | --- | --- | --- | --- | --- | --- | --- | --- | --- | --- | --- | --- | --- | --- | --- | --- | --- |
|  |  | LD n=4 | | | | Mean ± SD | LDA n=3 | | | Mean ± SD | *p* | LD n=3 | | | Mean ± SD | LDA n=3 | | | Mean ± SD | *p* |
| Inferior | p | 53,56 | 35,77 | 49,04 | 58,63 | 49,25±8,49 | 42,12 | 53,39 | 44,22 | 46,58±4,89 | 0,70 | 9,69 | 43,33 | 44,53 | 32,52±16,15 | 43,02 | 42,77 | 42,12 | 42,64±0,38 | 0,426 |
|  | mp | 54,61 | 49,39 | 50,31 | 54,92 | 52,31±2,48 | 46,17 | 44,27 | 51,06 | 47,17±2,86 | 0,08 | 16,4 | 43,52 | 38,97 | 32,96±11,86 | 45,82 | 45,49 | 44,56 | 45,29±0,53 | 0,216 |
|  | mc | 51,66 | 40,20 | 51,03 | 51,29 | 48,55±4,82 | 48,06 | 52,06 | 53,43 | 51,18±2,28 | 0,49 | 8,50 | 45,71 | 19,38 | 24,53±15,62 | 53,26 | 45,27 | 43,27 | 47,27±4,32 | 0,118 |
|  | c | 48,67 | 46,37 | 56,99 | 54,41 | 51,61±4,27 | 50,19 | 55,67 | 49,31 | 51,72±2,81 | 0,97 | 14,03 | 44,34 | 30,64 | 29,67±12,39 | 51,28 | 45,91 | 42,75 | 46,65±3,52 | 0,136 |
| Superior | p | 23,58 | 37,61 | 42,41 | 50,37 | 38,49±9,74 | 47,73 | 43,23 | 44,17 | 45,04±1,94 | 0,38 | 10,75 | 33,59 | 31,94 | 25,43±10,40 | 38,25 | 44,96 | 37,61 | 40,27±3,32 | 0,127 |
|  | mp | 22,37 | 41,69 | 38,67 | 47,73 | 37,62±9,39 | 34,04 | 43,99 | 37,72 | 38,58±4,11 | 0,89 | 12,17 | 19,24 | 30,2 | 20,54±7,42 | 40,49 | 33,85 | 43,03 | 39,12±3,87 | 0,035 |
|  | mc | 19,23 | 38,00 | 37,26 | 42,57 | 34,27±8,92 | 36,76 | 39,43 | 37,65 | 37,95±1,11 | 0,57 | 8,50 | 9,41 | 20,33 | 12,75±5,38 | 46,92 | 41,35 | 36,01 | 41,43±4,45 | 0,004 |
|  | c | 19,14 | 39,78 | 33,76 | 41,06 | 33,44±8,70 | 41,28 | 40,45 | 39,95 | 40,56±0,55 | 0,29 | 8,60 | 14,73 | 19,00 | 14,11±4,27 | 43,81 | 37,18 | 42,24 | 41,08±2,83 | 0,002 |
